# Infective Jaundice

**Published:** 1917-11

**Authors:** 


### MEDICAL

***Infective Jaundice.*** By Sir Bertrand Dawson, Col. A. M. S., Physician, To H. M. the King, Consulting Physician British Expeditionary Forces; W. E. Hume, Lt. Col. R. A. M. C. (T. F.); and S. P. Bedson, Capt. R. A. M. C. Physician London Hospital. Abstracted from the *British Medical Journal*, Sept. 15, 1917.

(Note also in this connection the article on "Spirochaetes in the Urine", p. 54.)

The authors cite the ancient history of epidemic jaundice and its recent recognition by workers from various parts of the world.

The onset of infective jaundice is characterized by a sudden attack of headache, vomiting, and diarrhea, accompanied by a feeling of intense weakness. Temperature is usually high. Blood appears early in stools. Hemoptysis is frequently present. The jaundice usually appears about the fifth day after the onset of the attack, and begins to fade about the eighth or ninth day.

Examination usually reveals an enlargement of the liver as an accompaniment of intense jaundice. The blood shows secondary anemia and polymorphonuclear leucocytosis. In the urine appear large quantities of bile and considerable albumin with numerous casts. The Spirochaeta icterohaemorrhagiae may be found early in the blood, and later in large numbers in the urine — more commonly after the second week. Herpes labialis, which is hemorrhagic, appears in at least 40 o/o of all cases. The pulse rate remains low in proportion to the temperature. Most of the severe cases show evidence of acute bronchitis. Occasionally there is a secondary rise of temperature at about the beginning of the third week, without exascerbation of symptoms. Most cases recover fully. Mortality does not exceed 4 or 5 o/o.

Treatment is purely symptomatic. Salvarsan has proved of no avail. Diagnosis in the early stages of the disease may be verified by demonstrating the spirochaete in the peripheral blood stream. Injection of infected human blood or urine into the peritoneal cavity of the guinea pig may produce a characteristic illness in that animal, the incubation period being at least six days.

The authors believe that many cases of fever of unknown origin are due to this infection, as jaundice is not always present, and the diagnosis without this symptom may be difficult.

Experimental pathology shows the spirochaete — discovered by Inada and Ido in 1914 — the causative organism. The infection is probably spread by urine and faeces. The field rats, which, it is reported, may frequently be carriers, may act as a reservoir for the dissemination. No intermediate parasitic host has been determined.

Details of clinical and experimental pathology are given. A complete bibliography is appended.

